# Participants’ Roles in Bullying Among 7–11 Year Olds: Results from a UK-Wide Randomized Control Trial of the KiVa School-Based Program

**DOI:** 10.3390/bs15020236

**Published:** 2025-02-19

**Authors:** Judy Hutchings, Ruth Pearson, Malavika Babu, Suzy Clarkson, Margiad Elen Williams, Julia R. Badger, Rebecca Cannings-John, Richard P. Hastings, Rachel Hayes, Lucy Bowes

**Affiliations:** 1 Centre for Evidence Based Early Intervention, Bangor University, Bangor LL57 2DG, UK; ruth.pearson04@outlook.com (R.P.); s.clarkson@bangor.ac.uk (S.C.); margiad.williams@bangor.ac.uk (M.E.W.); 2 Centre for Trials Research, Cardiff University, Neuadd Meirionnydd, Heath Park, Cardiff CF14 4YS, UK; babum@cardiff.ac.uk (M.B.); canningsrl@cardiff.ac.uk (R.C.-J.); 3 Department of Education, University of Oxford, Oxford OX1 2JD, UK; julia.badger@education.ox.ac.uk; 4 School of Social Policy and Society, University of Birmingham, Birmingham B15 2TT, UK; r.hastings@birmingham.ac.uk; 5 Department for Public Health and Sports Sciences, Faculty of Health and Life Sciences, South Cloisters, St Luke’s Campus, University of Exeter, Exeter EX1 2LU, UK; r.a.hayes@exeter.ac.uk; 6 Department of Experimental Psychology, Oxford University, Oxford OX2 6GG, UK; lucy.bowes@psy.ox.ac.uk; 7 National Institute for Health and Care Research (NIHR), Oxford Health Biomedical Research Centre, Oxford University, Oxford OX3 7JX, UK

**Keywords:** bullying, victimization, children, intervention, KiVa, school, participant roles

## Abstract

This paper describes the social architecture model of school-based bullying behavior. The model proposes that the behavior of all students affects rates of bullying. Alongside self-reported victims and bullies, the model identified four bystander roles: assistant, reinforcer, outsider, and defender. The level of support for bullies varies based on school policies that address bullying and promote school connectedness. The universal components of the KiVa school-based anti-bullying program designed to teach pupils to stand against bullying are described. The Stand Together trial, a UK-based randomized controlled trial, recruited 11,000+ students from 118 schools across the UK, half of whom received the KiVa program whilst the remainder delivered usual practice to address bullying. The main trial results reported a significant reduction in victimization in favor of KiVa. This paper examines data collected on the pupil-reported Participant Role Questionnaire (PRQ), one of the secondary measures used to explore whether significant reductions in victimization were accompanied by changes in bystander behavior. The results showed reductions in the student response rates of self-identified roles as bullies, assistants, and reinforcers in favor of KiVa, but outsider roles increased, and defender roles reduced. This provides tentative support for the social architecture model as taught in the Stand Together KiVa trial but also suggests that further work needs to be conducted to support the development of defender behaviors and address this important public health challenge.

## 1. Introduction

School-based bullying is a major international health concern requiring action at a policy level, in the wider community, and in schools (Iati, 2019; Srabstein & Leventhal, 2010; Currie et al., 2012) In the UK, schools are required to have anti-bullying policies, although there is considerable variability in terms of their content and the ways in which schools implement them (Smith et al., 2012, 2008).

The most widely used definition of bullying (Olweus, 1994) has three components: intention, repetition, and the involvement of a higher status of individual or individuals.

*“a student is being bullied or victimized when he or she is exposed, repeatedly and over time, to negative actions on the part of one or more other students” (and where there is) “an imbalance of strength (an asymmetric power relationship)”.*

(Olweus, 1994, p. 1173)

Experience of bullying in school, for both victims and perpetrators, can have significant negative effects in childhood and through adolescence and is linked to a range of difficulties into adulthood (Camodeca & Nava, 2020; Ttofi et al., 2011; Rigby, 2003; Smith, 2016; Wolke & Lereya, 2015; Arseneault et al., 2010) with evidence of different effects on victims than perpetrators (Brown & Taylor, 2008; Wolke et al., 2013). The impact of victimization is associated with depression and anxiety (Arseneault et al., 2006; Eastman et al., 2018; Ttofi et al., 2011; Zhang et al., 2020), poor academic achievement, sleep disturbances, somatic and psychosomatic complaints, night terrors, the loss of self-esteem, absenteeism, poor life satisfaction, loss of appetite, poor physical health (Fisher et al., 2017; Hawker & Boulton, 2000; Smith & Brain, 2000; Smith, 2016; Wang, 2023), and thoughts of suicide and suicide attempts in later life (Moore et al., 2017; Ttofi et al., 2011). Perpetrating bullying can also contribute to detrimental short- and longer-term difficulties (Smith, 2016; Grennan & Woodhams, 2007; Zuckerman, 2016). This can include an increased risk of low academic attainment, employment difficulties, lower socioeconomic status, a higher risk of being involved in domestic violence (Brown & Taylor, 2008), and involvement in criminal behavior in young adulthood (Fergusson et al., 2014). Those students who assist bullies by joining in but not initiating bullying can also experience an increased risk of aggressive externalizing problems, low academic achievement, involvement in criminal activity, and substance misuse (Clark et al., 2022; Evans et al., 2019; Riffle et al., 2021). Even witnessing bullying may contribute to subsequent depressive and anxiety symptoms (Camodeca & Nava, 2020; Midgett & Doumas, 2019).

The school environment contributes to bullying perpetration, with bullying norms playing a large role in rates of school bullying. School connectedness matters and can be defined as the cohesiveness between diverse groups in the school community, including students, families, school staff, and the wider community (Rowe et al., 2007). Feelings of school connectedness have a positive impact on children (McNeely et al., 2002), and are associated with lower levels of school bullying (Espelage et al., 2014; Glew et al., 2005; Merrin et al., 2018), whereas insufficient adult supervision, inadequate anti-bullying policies, and/or a permissive attitude toward bullying within a school setting are associated with an increased risk of bullying behaviors (Liu et al., 2020; Espelage & Swearer, 2003). Types of bullying have been categorized in different ways. Olweus (1993) describes bullying as a sub-form of aggressive behavior, which he classified as direct and indirect (Olweus, 1993), with direct identification being verbal and physical. Other researchers, e.g., Orpinas and Horne (2006), identified three categories—direct, indirect, and relational or psychological—which include damaging or vandalizing possessions, writing threatening or frightening notes, and social isolation. Other classification systems have been suggested, including the addition of sexual bullying (Pepler et al., 2006). Salmivalli and colleagues identified nine categories of bullying: physical, verbal, social, cyber, social manipulation, material, threat, racist, and sexual (Menesini & Salmivalli, 2017; Salmivalli et al., 2011a; Smokowski & Kopasz, 2005). However, as many as 25% of teachers often do not recognize some types of bullying (Boulton, 1997) with teachers being more likely to identify physical bullying incidents than relational ones (Chen et al., 2018) and several indirect forms of bullying are often dismissed (Maunder et al., 2010) Consequently, teachers also need help to both recognize and intervene effectively when bullying occurs (Ey & Campbell, 2022; Campbell et al., 2019).

### 1.1. Roles in Bullying

Bullying is increasingly recognized as a group process with all students contributing to the maintenance, escalation, and potential of the reduction in bullying in schools (Salmivalli et al., 1996; Salmivalli, 2001; Twemlow et al., 2004; Goodstein, 2013; Twemlow & Sacco, 2013; Swearer & Hymel, 2015). Alongside bullies and victims, including bully victims, children who both bully and are bullied, Salmivalli et al.’s (1996) social architecture model describes a range of bystander roles, suggesting that bullying behaviors provide social reward for the perpetrator by fulfilling a need such as social attention, and improved social status among, or power over, their peers (Salmivalli et al., 1996, 2011b; Saarento & Salmivalli, 2015). The presence and behavior of others during a bullying incident can reinforce the bullying behavior by actively joining in, laughing, or watching, or conversely reducing its occurrence by removing the social reward. Salmivalli et al. (1996) identified four bystander roles that influence the bullying situation: being an assistant to the bully, the reinforcer of the bully, the outsider, and the defender of the victim (Salmivalli et al., 1996; Salmivalli et al., 2011b). Assistants take an active role, but they follow the perpetrator’s lead and/or take instruction from them. They do not initiate the attack but are seen by victims as bullies (Salmivalli et al., 1996). Reinforcers provide social support for the bully by laughing or shouting encouragement. Their actions can have an adverse effect on the victim, leaving them feeling socially isolated and lowering their self-esteem (Salmivalli et al., 1996). Outsiders remain neutral, which may be due to the fear of becoming a target themselves but can be seen by victims and others as approving of the bullying by being silently complicit (Salmivalli et al., 1996). Defending can take different forms, including confronting the bully (bully-oriented defending) and comforting the victim (victim-oriented defending) (Reijntjes et al., 2016). Their support by telling the bully to stop, letting an adult know what is happening, comforting the victim, or other supportive behaviors reduce the victim’s feeling of social isolation (Salmivalli et al., 1996). Knowledge of the way in which bystander behavior impacts school-based bullying guided the development of the KiVa school-based bullying program (Espelage et al., 2013; Guo, 2021). There is evidence that students in KiVa schools perceive a significant increase in the number of classmates acting as defenders of victims and also see an increase in their own defending behavior (e.g., Saarento & Salmivalli, 2015). Less is known about changes in other participants’ roles that may influence bullying.

### 1.2. Kiva in Finland

Finnish comprehensive schools provide for children aged 7 to 15 and are non-selective, and Finish children perform very highly in terms of academic outcomes (Niemi, 2023). As in UK schools, Finnish schools are required to have an anti-bullying policy, but over a 10-year period this had no impact on reported rates of bullying (Salmivalli et al., 2011a). Professor Salmivalli had, for many years, been undertaking research on bullying and as a result, in 2006, the Finnish government funded her and her team at the University of Turku to develop a program for schools. The program, KiVa, has all the tools needed to address bullying, and has achieved extremely good outcomes in terms of reduced reported bullying and victimization in Finland. Following the success of the original randomized controlled trial (Kärnä et al., 2011), KiVa was offered to all Finnish comprehensive schools, achieving a 90% take up and showing year-on-year reductions in reported bullying over a seven-year period (Herkama et al., 2017).

### 1.3. The Components of the KiVa Program

KiVa has a set of universal school-wide activities to impact the behavior and attitudes of all students and a specific targeted intervention strategy to address bullying (the indicated action plan).

The universal component is as follows:

- Staff materials, including a PowerPoint presentation, are provided to ensure that all members of school staff are familiar with the definition of bullying, the program, and the process of referral to the KiVa team.
- Materials to be shared with all parents, including a presentation for a launch meeting with parents, are provided. Parents are informed that the school is a KiVa school and stands against bullying and are also provided with a booklet that describes ways to talk with their child about bullying.
- Staff supervise break times and lunch times and wear a hi-visibility vest with a KiVa symbol on it to remind students that they are in a KiVa school.
- Posters are distributed throughout the school to remind everyone that they are in a KiVa school and that they have a responsibility to make the school a happy place.
- KiVa has three sets of lesson units for ages 7 to 9 (Unit 1), 10 to 12 (Unit 2), and 13 to 15-year-olds (Unit 3). For units 1 and 2, there are ten 90 min lessons. Lessons are scripted and include videos, small and large group discussions, role-play, written assignments, and other play activities and KiVa games. Lessons start with components to promote prosocial behavior, learning to identify emotions, how to be a member of the team, and how to make the classroom a happy place. As they progress throughout the year, students and their teachers address issues related to bullying, how to define it, how to recognize it, and safe ways to stand against it. Throughout the year, students identify the KiVa rules. The aim is to encourage students away from behaviors that support bullying and towards behaviors that promote defense of victims.
- The annual anonymous student survey enables children to report, annually, on their own behavior in terms of whether or not they have experienced or perpetrated bullying. The overall result is reported back to their school, along with information regarding the responses of KiVa schools in general.

The indicated action component is as follows:

This is used when a confirmed bullying incident is identified. To achieve this, every member of the school staff is taught the definition of bullying and what to do if they believe that bullying may have taken place. The incident is recorded and sent to the trained KiVa Team within the school. The team reviews the information and decides whether it meets the criteria to be considered bullying. If it does not, it is dealt with through normal school procedures for resolving disagreements, fallouts, accidents, and misunderstandings. If it is identified as a bullying incident, there is a structured process for the KiVa Team to follow. This does not require blaming the bully but the commitment to finding a solution; the no-blame approach has been shown to achieve equally good results, particularly with the younger age range. As a result of this process, 78% of incidents ceased altogether, and a further 19.5% were reduced; in other words, it has an impact on 97.5% of all cases addressed (Garandeau et al., 2014).

### 1.4. KiVa in the UK

UK schools are required to have an anti-bullying policy, but, as in Finland before the introduction of KiVa, it is left to individual schools to decide what goes into it and how to implement it (Smith et al., 2012), and no specific evidence-based advice is provided.

Whilst the results from Finland are very convincing, the system of education in the UK is very different from that of Finland and, therefore, evidence of transportability was required. An analysis of the required curriculum for compulsory personal social education in both England and Wales showed that the KiVa curriculum covered 50% of the required topics, and in 2013, with the Welsh Government’s support, KiVa was introduced in Wales. Promising reductions in bullying-related behavior were reported from the first 17 schools to implement the program (Hutchings & Clarkson, 2015). At that point, a license to disseminate KiVa across the UK was obtained from Finland, and it was implemented in the first 41 UK schools to trial the program (Clarkson et al., 2019) and again reported significant reductions in both bullying and victimization at a reasonable ongoing implementation cost. Following the Hutchings and Clarkson (2015) and Clarkson et al. (2019) papers, both reporting significant benefits from early implementer schools, a small lottery grant was obtained for a pilot RCT with 22 schools. This did not demonstrate significant benefits but reported considerable variability in its implementation across the schools (Axford et al., 2020).

### 1.5. The Stand Together Trial

Based on the initial pre-post evidence that KiVa reduced victimization and bullying in UK primary schools (Hutchings & Clarkson, 2015; Clarkson et al., 2019), in 2019, the National Institute for Health and Care Research (NIHR) funded a randomized controlled trial (RCT), the UK Stand Together Trial, to establish outcomes based on one year of KiVa delivery compared to usual practice. With collaboration across five universities (Bangor, Oxford, Exeter, and Warwick for recruitment and Cardiff for data analysis), 118 state-maintained primary schools and over 11,000 students were recruited from across four areas (North Wales, Oxfordshire, Devon, and Birmingham). Each of the four sites recruited 28 or 29 schools. Parents were given an opt-out option for their child’s participation. Children provided their written assent via e-tablets for self-reported outcomes at each data collection point. From a potential of 11,922 students, 11,111 (93.2%) students completed a baseline questionnaire. Child questionnaires were administered via e-tablets, with researchers reading the questions aloud to the whole class. Further information about recruitment and methods is reported in the main outcome paper (Bowes et al., 2024). Rates of free school meals (FSMs) ranged from 7 to 28%, and the number of 7–11-year-old pupils ranged from 64 to 224. Schools were randomized on a 1:1 basis, taking into account school size, rurality, and the level of free school meal eligibility. Data were collected on levels of victimization using the self-reported Olweus Bully Victimization Questionnaire (OBVQ, Olweus, 2006). Secondary outcomes included self-reported bullying (OBVQ), empathy towards victims (Pöyhönen et al., 2010), and roles in bullying situations as measured using the “Participant Role Questionnaire” (PRQ) (Salmivalli & Voeten, 2004). Teachers rated children based on the Teacher Strengths and Difficulties Questionnaire (TSDQ), which records behavioral and emotional problems as well as pro-social behavior. They also collected data on lesson administration. At baseline, 21.6% of students reported being bullied in the usual-practice group and 20.3% in the KiVa-intervention group, which were broadly comparable with reports from other studies.

Parental consent was not required for children to participate in KiVa school-based activities as it formed part of the compulsory Personal Social Educational curriculum in both England and Wales. The trial was funded to undertake the evaluation of KiVa, which was delivered by randomized intervention schools whilst control schools continued with the usual anti-bullying practice. Ethical approval for the collection of trial data was obtained from the Bangor University Psychology Research Ethics and Governance Committee (reference: 2019-16592). This included giving parents an option to remove their children from the collection of trial data.

All analyses were pre-registered and performed blind by statisticians at the Centre for Trials Research, a UKCRC Registered Trials Unit at Cardiff University. Details on recruitment, data collection, statistical analysis procedures, and hypotheses were reported in a protocol paper (Clarkson et al., 2022).

### 1.6. Training and Support for KiVa Trial Schools

Funding was obtained for schools to deliver KiVa, and in order to address some of the implementation challenges experienced during the Axford et al. (2020) trial, KiVa trainers were appointed for each site. During the trial year, trainers met each term with the schools and were also available to schools for consultation if requested. Two school staff from each allocated KiVa school attended a two-day training coordinated through the Children’s Early Intervention Trust Charity (CEIT) that holds the UK KiVa dissemination license. At the KiVa training session, the roles of the school coordinator and KiVa team lead were explained, and the participants planned for the launch of Kiva in their school. The KiVa coordinator was responsible for ensuring that all school staff were aware of the definition of bullying, how to recognize it, and what to do when it is identified. They also oversaw the implementation generally, ensuring that class teachers delivered the KiVa lessons and oversaw the annual online KiVa pupil survey. The KiVa team lead was responsible for training identified staff as members of the KiVa team that was set up to address confirmed bullying incidents.

Despite considerable disruption in schools returning from the COVID-19 shutdown and with high levels of post-COVID-19 absenteeism, the UK Stand Together KiVa RCT results (Bowes et al., 2024) showed that after one year of implementation, the KiVa program had significantly reduced the odds of bullying victimization among students by 13% compared with students from schools continuing with usual practice. In addition, KiVa students demonstrated significantly higher levels of empathy, and fewer teachers reported peer relationship problems. The defender scale of the Participant Role Questionnaire (PRQ), reported as a secondary outcome, indicated that students in the KiVa arm had 24% lower odds of defending bullying compared to the usual-practice arm (significant at the *p* = 0.03 level). Exploratory findings indicated that the effectiveness of KiVa was consistent across different levels of the school’s socioeconomic status, school size, and gender, and the economic evaluation reported that KiVa was a low-cost intervention compared to many other UK school-based interventions. Together, these results suggest that KiVa is a feasible and efficient strategy for reducing bullying in schools.

The positive outcome from the trial in favor of KiVa provided the opportunity to explore possible changes in children’s responses to the Participant Role Questionnaire (PRQ) (Salmivalli et al., 1996).

### 1.7. Aims

The PRQ was included in the main RCT as one of the exploratory objectives. The PRQ data provide an opportunity to establish whether the anticipated participant roles changed, with reductions in assisting, reinforcing, and being an outsider and increases in defending, occurring, as implied by the social architecture model (Salmivalli et al., 1996).

## 2. Methods

School recruitment is described in the Clarkson et al. (2022) protocol paper and the Bowes et al. (2024) main results paper. It involves circulating information about the trial in each of the trial areas and recruiting mainstream primary schools with at least two classes of pupils aged between 7 and 11 on a first-come-first-served basis. One hundred and eighteen schools and 11,922 students were recruited, either 29 or 30 schools, at each of the four sites. Baseline questionnaires were completed by 11,111 (93.2%) students aged between 7 and 11 years, and 9981 (90.1%) completed follow-up questionnaires one year later, including 5321 (90.0%) students in the KiVa schools and 4660 (90.2%) in usual-practice schools. Questionnaires were administered in person using e-tablets, with researchers reading the questions and answering options aloud to the whole class. The KiVa program was implemented in the academic year September 2021 to July 2022, and survey outcomes were measured between April and June 2022. Baseline student and teacher characteristics were well balanced across the trial arms.

### 2.1. Materials

The PRQ (Salmivalli et al., 1996) provides a measurement of the multifaceted roles of bullying, which range from direct participation to defending. An accurate assessment of these roles is critical for developing targeted interventions. In its initial form, the PRQ was a peer-report measure. Initially comprising 50 items, it has since undergone adaptations, with subsequent versions including the 15-item version (Demaray et al., 2016), which was developed to address concerns about the length and complexity of the original questionnaire. The 15-item version preserves the core structure and includes the original five roles: the bully, assistants to the bully, reinforcers of the bully, outsiders, and defenders of the victim. The PRQ has been shown to have good internal consistency with Cronbach’s alpha coefficients of 0.93 for the bully scale, 0.95 for the assistant scale, 0.90 for the reinforcer scale, 0.89 for the defender scale, and 0.88 for the outsider scale (Salmivalli et al., 2011b).

Although developed as a peer-report measure, the PRQ was subsequently revised as a self-report measure, in part due to ethical concerns about asking students to nominate/report on their peers (Espelage & Holt, 2001; Verlinden et al., 2015) and there were concerns from both teachers and researchers about parent opt-out from peer ratings, allowing pupils to rate others as bullies, which might significantly reduce recruitment. The self-report version involves participants reporting on their own behavior and experiences (Felix et al., 2011) and has become a preferred approach due to its time and cost-effectiveness (Espelage & Swearer, 2003; Furlong et al., 2010; Vivolo-Kantor et al., 2014). In Salmivalli et al.’s (1996) study, both peer nominations and self-report versions of the PRQ were collected, and the self-estimated scores on the bully, assistant, reinforcer, outsider, and defender scales were positively correlated with the corresponding peer-estimated scores.

The 15-item self-report version was used in the current trial. In the self-report version, the wording and language of the items ask only how the person completing the survey behaves. Each of the 15 items is rated on a 3-point Likert scale (0 = never, 1 = sometimes, 2 = often). An extra response was added to all questions in the Stand Together trial to give students the option to not answer the questions as required for ethical approval (3 = I’d rather not say).

- Bully scale: “I bully other children”, “I make other people bully other children with me”, and “I always find new ways to annoy the bullied pupil”. Higher scores indicate more frequent bullying behavior (negative).
- Assistant scale: “I join in with bullying when someone else starts it”, “When someone is bullying other children, I help the bully”, and “When someone is bullying other children, I help them, maybe by catching the bullied child”. Higher scores indicate more frequent assistant behavior (negative).
- Reinforcer scale: “When someone is being bullied, I go and see what’s happening”, “When someone is being bullied, I laugh”, and “When someone is being bullied, I tell the bully to keep on doing it”. Higher scores indicate more frequent reinforcer behavior (negative).
- Outsider scale: “I am around when other people get bullied”, “When someone is being bullied, I stay away from where it is happening”, and “When someone is being bullied, I don’t take sides with either the bully or the pupil being bullied”. Higher scores indicate more frequent outsider behavior (positive).
- Defender scale: “When someone is being bullied, I try and make the bullied pupil feel better and say they should tell a teacher”, “When someone is being bullied, I tell the bullies to stop”, and “I try and make the bullies stop bullying other people”. Higher scores indicate more frequent defender behavior (positive).

The PRQ was administered as part of a group of measures completed by children and included the 22-item version of the Olweus Bullying and Victimization Questionnaire (OBVQ; Olweus, 2006), which provided data for the main hypothesis regarding reduction in victimization (see Clarkson et al., 2022; Bowes et al., 2024).

### 2.2. Statistical Analysis

Pupils’ self-reported results on the bully, assistant, reinforcer, outsider, and defender scales were assessed by averaging scores from the corresponding three items of the PRQ to a single measure. Each subscale was categorized into two categories for analysis (never = 0 if the subscale score was zero; sometimes/often = 1 if the subscale score was greater than zero) and reported as frequencies alongside percentages. A logistic regression model was used to assess the impact of KiVa training on the PRQ scales. Results were presented as the odds ratio (ORs) alongside the 95% confidence interval (CI). All analyses were intended to treat without imputation (a complete case analysis restricted to students with responses at both baseline and follow-up), with outcomes compared between KiVa and usual-practice groups using three-level regression models (allowing for clustering between students within school and between schools within sites). Analyses controlled for school-level stratification variables (school size, proportion of students eligible for FSM), key student characteristics (age, sex), and baseline outcome measures. The statistical analyses took into account clustering by school and site using random effects. All analyses controlled for the school-level stratification variables (site, school size, proportion of children eligible for FSM), the age and gender of the pupils, and the baseline measure of the outcome. Data analysis was conducted in IBM SPSS Statistics version 26 (IBM Corporation, Armonk, NY, USA), R software (R Core Team (2021)), and Stata^®^ version 17 (StataCorp LP, College Station, TX, USA).

## 3. Results

One hundred and eighteen schools were recruited across the four trial sites between October 2020 and February 2021, with 11,111 students (5944 KiVa arm; 5167 usual practice) completing a baseline questionnaire (from a potential of 11,922 students). Twelve months of follow-up data were collected from 5321 (90.0%) students in the KiVa schools compared to 4660 (90.2%) in usual-practice schools. A total of 263 (KiVa) and 202 (usual practice) additional questionnaires were completed by students who had not completed a baseline questionnaire. Hence, the final numbers at 12 months were 5584 (KiVa) and 4862 (usual practice). The total number of students available for the complete case analysis (baseline and 12-month follow-up) was 9981. Student characteristics recorded at baseline were similar between arms; 49% of students were female, and the majority self-reported their age as being between 8 and 10 years of age (Table 1).

### 3.1. The PRQ Bully Scale

Most respondents reported never having bullied other children at both baseline and 12-month follow-up; however, for those that did report bullying other children, there was a greater reduction in the KiVa arm at 13.9% to 9.7% and a reduction of 4.2 percentage points (Table 2) compared with a reduction of 0.6 percentage points (from 12.7% to 12.1%) in the usual-practice arm. When rates of bullying at 12 months follow-up were compared between arms, the adjusted odds ratio (AOR) was 0.72 (95% confidence interval (CI) = 0.53 to 0.99, *p*-value 0.041), indicating a statistically significant 13% reduction in the odds of victimization among students attending schools randomized to receive KiVa when compared to those attending schools receiving usual practice.

### 3.2. The PRQ Assistant Scale

The rates of sometimes/often assisting a bully were comparable at baseline (18.6% KiVa vs. 18.5% usual practice). The KiVa arm experienced a reduction in assistance of 2.1 percentage points over the 12 months (from 18.6% to 16.5%), with usual-practice schools experiencing an increase of 2.1 percentage points (from 18.5% to 20.6%). When rates of assistance at 12 months follow-up were compared between arms, the AOR was 0.79 (95% CI = 0.62 to 0.99, *p*-value 0.046), indicating a statistically significant lower rate of assistance among the students attending schools that were randomized to receive KiVa, when compared to students attending schools receiving usual practice.

### 3.3. The PRQ Reinforcer Scale

Higher rates of reinforcing bullying were observed across both arms at baseline (80.9% KiVa vs. 79.7% usual practice), with a small decrease in the KiVa arm of 1.5 percentage points and a small increase of 1.3 percentage points in children sometimes or often reinforcing bullying in the usual-practice arm. The difference of 1.4% between arms did not achieve statistical significance (AOR = 0.90 (95% CI = 0.73, 1.12), *p* = 0.358).

### 3.4. The PRQ Outsider Scale

The majority of children from both trial arms reported being an outsider sometimes or often at baseline (94.2% KiVa vs. 93.6% usual practice). At follow-up, the percentage of children reporting being an outsider to bullying increased by 1 percentage point in the KiVa arm and by 2.5 percentage points in the usual-practice arm. The difference between arms at follow-up of 0.9 percentage points achieved statistical significance (AOR = 0.73 (95% CI = 0.59, 0.90), *p* = 0.003).

### 3.5. The PRQ Defender Scale

The defender scale was reported as a secondary outcome in the main outcomes paper (Bowes et al., 2024). A high proportion of children reported defending bullying at baseline (97.9% KiVa vs. 97.5% usual practice), which reduced over time. There was evidence to suggest a difference between the arms at follow-up (AOR 0.76 (95% CI 0.59 to 0.97), *p* = 0.03).

## 4. Discussion

The KiVa curriculum was built on the social architecture model (Salmivalli et al., 1996) with the aim of the universal components being to change the responses of all children to bullying. The Stand Together trial was successful at achieving its main outcome of reducing victimization (Bowes et al., 2024). The present paper explored students’ responses to the Participant Role Questionnaire (PRQ) to see whether, and if so how, the significant reduction in victimization reported for the KiVa arm of the UK Stand Together trial (Bowes et al., 2024) was reflected in the responses of students to other bullying roles.

All schools in the UK are required to have an anti-bullying policy and to address cases of bullying, and this forms part of the compulsory PSE curriculum for UK schools. The main trial result is important, particularly given that it was achieved during the post-COVID-19 return to school period in a year that had significant teacher and pupil absenteeism. However, addressing bullying is an ongoing process. In Finland, the program showed year-on-year reductions in victimization over a seven-year period when, following the results from the initial randomized controlled trial, the Finnish government rolled the program out across Finland (Kärnä et al., 2011; Herkama et al., 2017).

Having established that the program was effective in reducing bullying in UK primary schools, the data from the PRQ enabled us to explore whether the UK KiVa intervention significantly reduced participant roles associated with increased bullying (bullying, assisting, reinforcing, and outsiders) and increased those intended to help reduce bullying (defending). Whilst most children are not involved in bullying as victims or bullies, the social architecture model implies that all children have a role to play in bullying. Our results provide tentative support for this model, demonstrating some changes in the responses of all children to the PRQ, not just those directly involved in bullying.

The OBVQ reduction in bullying behavior, based on a single question reported in the main trial paper, favored KiVa but did not achieve significance (*p* = 0.08; Bowes et al., 2024). However, the PRQ bullying response, based on three questions, demonstrated a significant reduction in bullying relative to usual-practice schools (*p* = 0.041), providing evidence that the reductions in the odds of victimization (13%, Bowes et al., 2024) were accompanied by a reduction in the self-reported rates of bullying alongside the significant reported reduction in victimization.

The result for the assistance scale—students who do not initiate bullying but join in when it is occurring—showed an increase in never assisting for the KiVa condition and a decline in the usual-practice condition (*p* = 0.046) and suggested that KiVa worked in the way intended.

The results from the reinforcer scale, which refers to students who stand by and contribute with laughter or other overt forms of support for the bully, also favored KiVa with the response of never reinforcing, reducing slightly in the usual-practice condition and increasing in the KiVa condition; however, this did not achieve significance (*p* = 0.358).

The result from the outsider scale showed that the number of students reporting sometimes or always being an outsider increased for both KiVa (94.2–95.2%) and usual practice (93.6 and 96.1%), but this increase was significantly greater in usual-practice schools than KiVa schools (*p* = 0.003).

The result for the defender scale showed that the high rates of baseline self-reported defender behavior (97.9% KiVa and 97.5% usual practice) reduced during follow-up (97% KiVa and 97.1% usual practice), and this was significantly greater in the KiVa condition.

Pupil self-reported bullying, assistance, and reinforcer ratings were all reduced, significantly in favor of KiVa in the case of bullying and assistance to bullies and non-significantly in the case of reinforcers. However, the increases in outsider behavior require explanation, particularly given that the reported rates of bullying and bully-supporting behaviors were reduced. The questions on the outsider scale report the actual behavior of outsiders at the time of bullying, “I am around when other people get bullied”, “When someone is being bullied, I stay away from where it is happening”, and “When someone is being bullied, I don’t take sides with either the bully or the pupil being bullied”. Their interpretation is not entirely clear as the motivation for behaving as if bullying is not happening can be varied, including a positive decision to avoid reinforcing bullying behavior or a fear of being bullied oneself. Whilst outsider behavior increased for the KiVa group, there was a significantly greater increase for the control children. Nevertheless, the fact that the result in the KiVa intervention arm was significantly less than the usual-practice arm is encouraging. However, given that the reported rates of defending behavior also reduced, the increase in outsider rates and reduction in defender behavior suggest that research and practice need to focus on how to support children to become active defenders and not outsiders. The added challenge that may have impacted this result was that data were collected before the end of the school year. In order to obtain data from the same class of children, the follow-up data were collected between March and June when, for some classes, only two-thirds of the class lessons were delivered. Initial lesson topics address working as a team, making the classroom a happy place, giving compliments, and learning to detect and understand feelings of self and others. Dealing with bullying comes later in the curriculum. This meant that some aspects of the program to address bullying would not have been delivered. Nevertheless, whilst there is material in the KiVa lessons designed to teach children how to do this, either by challenging bullies when it is safe to do so or by informing an adult or showing support to victims, these results may suggest that more work needs to be carried out in this regard.

### Limitations

In addition to the data collection timetable requiring that data were collected before the end of the school year, the post-COVID-19 year was affected by increased levels of both teacher and pupil absenteeism, and it is not known how many pupils were present when lessons were delivered. Absenteeism could have contributed to the lower levels of reduced victimization achieved due to reduced access/exposure to material for some children. Future trials should identify ways of collecting data on each child’s exposure to the KiVa lessons, such as through attendance registers for these lessons. The trial team now has access to the National Pupil Database, and analysis of pupil absenteeism will become available.

It is important to acknowledge that there is likely to have been significant variation in the delivery of bullying policies in usual-practice (UP) schools, as previously identified in other studies (Smith & Brain, 2000). The variation in school practice and lack of evidence-based guidance was one of the reasons for initially opting for the trial of KiVa in the UK. However, schools that registered for the trial are likely to be those that are more proactive in addressing bullying. Future papers (Bowes et al., 2025) should analyze and report on the data collected for the process evaluation on delivery in both KiVa and UP schools.

A limitation of the findings is that since the PRQ collects data on each question individually, students can report themselves as active on more than one of the scales, and further work is being undertaken to explore patterns of responses across the scales, which will provide an opportunity to establish the other response patterns of students who reported less support for bullies and more outsider and less defending behavior.

## 5. Conclusions

The trial took place during a very difficult post-COVID-19 period. The delivery of the program during the year 2021/22 was one in which teachers were struggling to address the challenges of students returning from lockdown and was also accompanied by significant absenteeism on the part of both pupils and teachers. Therefore, although the evidence suggests that the program was generally delivered as intended (Bowes et al., 2024), it is not known how much of the program individual students accessed. Nevertheless, despite these limitations, the evidence presented suggests that some changes in response to bullying fit with the social architecture model and show that the program influenced some behaviors in all children involved.

The findings add to the evidence for the importance of measuring bystander effects and implementing evidence-based whole-school approaches to address bullying. The results provide a valuable foundation for further research on the mechanisms by which KiVa changes rates of victimization, bullying, and the behavior of the whole school population. It has implications for longer-term integration and the embedding of KiVa into school practices.

## Figures and Tables

**Table 1:** Student characteristics.

	KiVa N = 5944	Usual Practice N = 5167
**Trial site** n (%)		
North Wales	1556 (57.5)	1148 (42.5)
Devon	1225 (52.6)	1106 (47.5)
Oxfordshire	1168 (50.2)	1161 (49.9)
Birmingham	1995 (53.2)	1752 (46.8)
**Student-reported characteristics**		
Females n (%)	2905 (49.0)	2560 (49.6)
Missing	10 (0.2)	1 (0.02)
**Age (in years)** n (%)		
6/7 years old	517 (8.9)	566 (11.0)
8 years old	1918 (32.9)	1650 (32.6)
9 years old	1913(32.8)	1702 (33.6)
10/11 years old	1417 (23.9)	1157 (22.4)
Missing	7 (0.12)	2 (0.04)

**Table 2:** Participant Role Questionnaire domains at baseline and 12-months follow-up by study arm.

	Baseline		12-Month Follow-Up				
	KiVa (N = 5944)	Usual Practice (N = 5167)	KiVa (N = 5584)	Usual Practice (N = 4862)	Trial Arm	Adjusted ^a^ OR (95% CI)	*p* Value
**Bully Scale ^b^, n (%)**							
Never	4541 (86.1)	4017 (87.3)	4640 (90.3)	3871 (87.9)	Usual Practice	Reference	
Sometimes/Often	735 (13.9)	586 (12.7)	499 (9.7)	535 (12.1)	KiVa	0.72 (0.53, 0.99)	0.041
**Assistant Scale ^c^, n (%)**							
Never	4340 (81.4)	3729 (81.5)	4255 (83.5)	3486 (79.4)	Usual Practice	Reference	
Sometimes/Often	995 (18.6)	846 (18.5)	841 (16.5)	902 (20.6)	KiVa	0.79 (0.62, 0.99)	0.046
**Reinforcer Scale ^d^, n (%)**							
Never	1026 (19.1)	947 (20.3)	1031 (20.4)	831 (19.0)	Usual Practice	Reference	
Sometimes/Often	4342 (80.9)	3722 (79.7)	4020 (79.6)	3535 (81.0)	KiVa	0.90 (0.73, 1.12)	0.358
**Outsider Scale ^e^, n (%)**							
Never	274 (5.8)	262 (6.4)	215 (4.8)	154 (3.9)	Usual Practice	Reference	
Sometimes/Often	4432 (94.2)	3864 (93.6)	4255 (95.2)	3808 (96.1)	KiVa	0.73 (0.59, 0.90)	0.003
**Defender Scale ^f^, n (%)**							
Never	108 (2.1)	116 (2.5)	148 (3.0)	127 (2.9)	Usual Practice	Reference	
Sometimes/Often	5173 (97.9)	4515 (97.5)	4784 (97.0)	4190 (97.1)	KiVa	0.76 (0.59, 0.97)	0.030

CI = confidence interval, OR = odds ratio. For all PRQ domains, Score 0 = never, Score > 0 = sometimes/always. ^a^ Adjusted for corresponding baseline measure, FSM and KS2 size, and age and sex of pupils. ^b^ Higher scores indicate more frequent bullying behavior (negative); ^c^ Higher scores indicate more frequent assistant behavior (negative). ^d^ Higher scores indicate more frequent reinforcer behavior (negative). ^e^ Higher scores indicate frequent outsider behavior (positive). ^f^ Higher scores indicate more frequent defender behavior (positive).

## Data Availability

The data presented in this study are available on request from the corresponding author (the data are not yet publicly available due to privacy and ethical restrictions).
